# Simple reaction time for broadband sounds compared to pure tones

**DOI:** 10.3758/s13414-016-1237-x

**Published:** 2016-11-22

**Authors:** Josef Schlittenlacher, Wolfgang Ellermeier, Gül Avci

**Affiliations:** 1 Institut für Psychologie, Technische Universität Darmstadt, Alexanderstraße, 10, 64283 Darmstadt, Germany; 2 Department of Psychology, University of Cambridge, Downing Site, Cambridge, CB2 3EB England

**Keywords:** Hearing, Psychoacoustics, Reaction time methods

## Abstract

Although many studies have explored the relation between reaction time (RT) and loudness, including effects of intensity, frequency, and binaural summation, comparable work on spectral summation is rare. However, most real-world sounds are not pure tones and typically have bandwidths covering several critical bands. Since comparing to a 1-kHz pure tone, the reference tone, is important for loudness measurement and standardization, the present work focuses on comparing RTs for broadband noise to those for 1-kHz pure tones in three experiments using different spectral and binaural configurations. The results of Experiments 1 and 2 yield good quantitative agreement with spectral loudness summation models for moderate and high sound pressure levels, measured using both pink noise covering almost the entire hearing range and bandpass-filtered pink noise with different center frequencies. However, at lower levels, the RT measurements yield an interaction of level and bandwidth, which is not in line with loudness scaling data. In Experiment 3, which investigated the binaural summation of broadband sounds, the binaural gain for white noise was determined to be 9 dB, which is somewhat larger than what had been found in previous RT measurements using 1-kHz pure tones.

## Introduction

Although spectral loudness summation is well understood, and despite the fact that it is important to consider when determining the loudness of real-world sounds, its quantification is still subject to discussion. Numerous experiments (e.g., Miller 1947; Pollack 1952; Zwicker 1958; Scharf 1959; Hellman 1985; Hellman and Zwicker 1987; Meunier et al., 2000; Schlittenlacher et al., 2011; 2015) studied several aspects of spectral loudness summation and yielded a wide range of results, which may explain that two different national standards, DIN 45631 (1991) and ANSIS34-2007 (2007), presently coexist. These two models differ by as much as 5 dB when predicting the loudness of broadband sounds (Fastl et al., 2009).

Such discrepancies among experiments arise because of the different stimuli, experimental setups, and procedures used. In particular, each of the psychophysical methods has its inherent advantages and problems. For example, the method of adjustment provides quite an easy task of matching two stimuli, however, the point of subjective equality depends on the order of the two sounds, an effect also known as the time-order error. By contrast, magnitude estimation without a standard requires only one stimulus to elicit a judgment on a given trial. However, it requires the participant to perform the mentally demanding task of direct scaling — another potential source of error when we are interested in perceptual rather than judgmental or context effects.

Simple reaction time (RT) avoids these disadvantages, simply requiring to press a button as soon as any sound is heard. Chocholle (1940) demonstrated that RT depends on loudness: the louder a sound, the faster the reaction to it. By proceeding to study the effect of frequency, and not just that of sound pressure level (SPL) as already Wundt (1874) had done, he could show that RT depends on loudness rather than physical intensity. Several RT studies have investigated further aspects on loudness since, for example binaural loudness summation (Chocholle 1944; Simon 1967; Schröter et al., 2007; Schlittenlacher et al., 2014; Lentz et al., 2014). They found RTs to monaural sounds to be slightly longer than those to binaural sounds, and that difference expressed in decibels quantitatively agreed quite well with that predicted by loudness models (e.g., Moore and Glasberg 2007). It should be pointed out, though, that there are also a few studies that postulate limits for RT being a correlate of loudness at low levels: Kohfeld et al., (1981) compared equal loudness-level contours with equal RT contours and found that the two differed at 40 phon and below. Epstein and Florentine (2006) measured RTs to 1-kHz and 4-kHz pure tones at very low sensation levels and concluded that RT “may not be the best tool to measure loudness at threshold”.

To our knowledge, Wagner et al., (2004) are the only ones who have explicitly addressed the relation between spectral loudness summation and RT. They asked six participants to react to the onset of a noise, which was presented over a large range of levels and had either a narrower or a wider bandwidth. In addition, they had their participants make loudness matches between the two noises. The level difference required for equal RT (LDERT) matched that required for equal loudness (LDEL) throughout the range of levels studied. In the context of evaluating the underlying architecture of RT models, Lentz et al. (2016) measured RTs to either one or two pure tones presented monaurally and at two different levels. As expected from a spectral gain in loudness, they reported shorter RTs to two tones compared to one tone at the same level, and the RTs highly correlated with calculated loudness level.

Typically, the effects of frequency, binaural summation, and spectral summation on RT were studied separately from each other. However, if RT is sought to be a measurement of loudness, it is important that also combinations of these effects have the same influence on RT as they have on loudness. The present study will focus on spectral summation and combine it with the other two effects. Furthermore, we will compare the RT to broadband noise with the RT to a pure tone. This comparison is particularly important for loudness measurements as the 1-kHz pure tone is the reference sound in the definition of loudness, and recent studies have shown that narrowband noises may have a different loudness than a pure tone at the same level and frequency (Hots et al., 2014). Experiment 1 expands the findings of Schlittenlacher et al., (2015), who evaluated the loudness of bandpass-filtered pink noise in different frequency ranges, but uses RT methodology.

Since the results of Experiment 1 turned out to be quite reasonable for moderate and higher levels but surprising for the lowest level studied, not showing any advantage in RT for the apparently louder broadband sounds compared to pure tones at 45 dB SPL, this interaction between sound pressure level and type of sound was sought to be examined in more detail in Experiment 2. Note that such an effect did not occur in the study by Wagner et al., (2004) and no such limit for RT being a correlate of loudness was shown in their study. However, they used two noises of different bandwidth rather than comparing a pure tone and a broadband noise. For this reason, Experiment 2 employs a 1-kHz pure tone versus a very broadband pink noise ranging from 200 Hz to 20 kHz.

Experiment 3 investigates the combination of spectral and binaural summation, i.e., whether the binaural gain for white noise is different from that obtained with a 1-kHz pure tone (see Schlittenlacher et al., 2014). Furthermore, Edmonds and Culling (2009) reported an effect of interaural correlation on the binaural loudness gain. The binaural LDEL was up to 2 dB larger for uncorrelated noise compared to diotic noise. Therefore, the present Experiment 3 uses both correlated and uncorrelated white noise to take this potential additional effect into consideration as well.

Altogether, the present work extends the investigation of reaction times to broadband noise by focusing on the combination with additional effects like the frequency range stimulated or binaural summation. It further emphasizes comparisons with a 1-kHz pure tone, the reference sound used in loudness modeling and standardization.

## General method

### Participants

Twenty listeners aged 20 to 27 years (median 22), 12 of them females and eight males, participated in Experiment 1, 21 in Experiment 2 (nine females, 12 males, 22 to 32 years, median 27) and 20 in Experiment 3 (15 females, five males, 19 to 29 years, median 22). All of them had thresholds in quiet better than 20 dB HL for frequencies between 250 Hz and 8 kHz, measured in octave steps and for each ear. Most of them participated for course credit, and participated in only one experiment, except for one who participated in Experiments 1 and 2 and another one who participated in Experiments 2 and 3.

### Apparatus and stimuli

In Experiments 1 and 2, the sounds were D/A converted by a RME Hammerfall DSP Multiface II audio interface and presented via Sennheiser HDA 200 headphones, being directly connected to the audio interface. Stimuli were free-field equalized in software by filtering the signals according to the data of Richter (2003) before storing them in the Waveform Audio File Format. In Experiment 3, the sounds were D/A converted by the same audio interface, but amplified by a Behringer HA8000 Powerplay Pro-8 and presented by Beyerdynamics DT-990 250 Ω headphones to the participants. The two different setups were used to ensure free-field equalized conditions for the comparison of broadband sounds to pure tones in Experiments 1 and 2 and to use the same setup as in earlier binaural studies (Schlittenlacher et al., 2014) in Experiment 3.

All experiments took place in a double-walled sound-proof chamber, manufactured by the Industrial Acoustics Company. For measuring reaction times, a custom-made telegraph key was used. It had a mechanical resistance comparable to that of a computer mouse. It was connected to a custom-made electronic timer, which was designed according to the prototype of Kerber (2008). The timer employs a high-precision clock rate of 1 ms and provides timing independently of the PC, transferring the recorded reaction times to the PC via USB.

All stimuli were generated digitally at a sampling rate of 48 kHz and at a resolution of 24 bit. They had Gaussian rise and fall times of 5 ms, the duration was 1 s in Experiments 1 and 2 and 200 ms in Experiment 3. For the bandpass-filtered noises, the slopes were steep enough to prevent loudness summation outside the defined band.

### Procedure

The participants were asked to press a telegraph key with their preferred hand as soon as they heard any sound. A trial started with the presentation of a red square on the screen for 200 ms. The foreperiod, following that warning signal and lasting until the onset of the sound, consisted of a fixed part having a duration of 500 ms and an additional variable part. For the latter, the duration was drawn randomly from an exponential distribution with a mean of 1 s. If the entire foreperiod had exceeded 5 s, the variable part was drawn randomly again. After pressing the telegraph key, the participants received visual feedback on the screen through a depiction of a button being pressed, telling them that their response had been registered. The interval between a reaction or end of the stimulus, whichever happened later, and the start of the next trial was 1.5 s.

Trials were arranged in blocks of about 100 trials to prevent fatigue. Between blocks, participants were allowed to take a break. Each block contained all conditions equally often and in random order. Before each experiment, participants completed a short training. In addition, each block started with two training trials.

Responses shorter than 100 ms or longer than 1 s were discarded from the analysis and the respective trial was repeated at a random position in the same block. For RTs less than 100 ms, we assume that the listener had anticipated the onset while RTs greater than 1 s are considered as misses of the onset. Such outliers were few in number, across all experiments about 0.3 % of all trials.

## Experiment 1: Bandpass-filtered pink noise

The subjective loudness evaluations of Schlittenlacher et al., (2015) showed the surprising result that a two-octave-wide bandpass-filtered pink noise around the most sensitive area of hearing was not considerably louder than one centered at the reference frequency of 1 kHz. Magnitude estimates were even a little higher for the latter. For this reason, the present experiment shall investigate whether reaction times confirm these rather surprising subjective evaluations or whether they agree with the predictions of loudness models. Furthermore, a 3.15-kHz pure tone is added to the set of stimuli. Doing so, the effects of frequency, bandwidth, and the interaction between the two can be studied.

### Stimuli and procedure

The four stimuli used were a 1-kHz pure tone, a 3.15-kHz pure tone, a bandpass-filtered pink noise with cutoff frequencies at 500 Hz and 2 kHz (mid-frequency noise), and a bandpass-filtered pink noise with cutoff frequencies at 1.25 kHz and 5 kHz (high-frequency noise). The two pure tones used represent the most sensitive frequencies within the ranges spanned by the two noises according to ISO 226 (2003). All stimuli were presented at three sound pressure levels: 45, 60, and 75 dB. According to DIN 45631 (1991), the 3.15-kHz pure tone has a loudness level being 6 phon higher than that of an equally intense 1-kHz pure tone throughout the range of levels studied. The effect of spectral summation across two octaves is predicted to be somewhat larger, with a difference in loudness level of ca. 10 phon between the 1-kHz pure tone and the mid-frequency noise. The combination of both frequency weighting and spectral summation leads to a predicted gain of ca. 14 phon for the high-frequency noise compared to the 1-kHz pure tone.

Each stimulus was presented 60 times to each participant, with data being collected in six blocks of 120 trials.

### Results

**Fig. 1:**
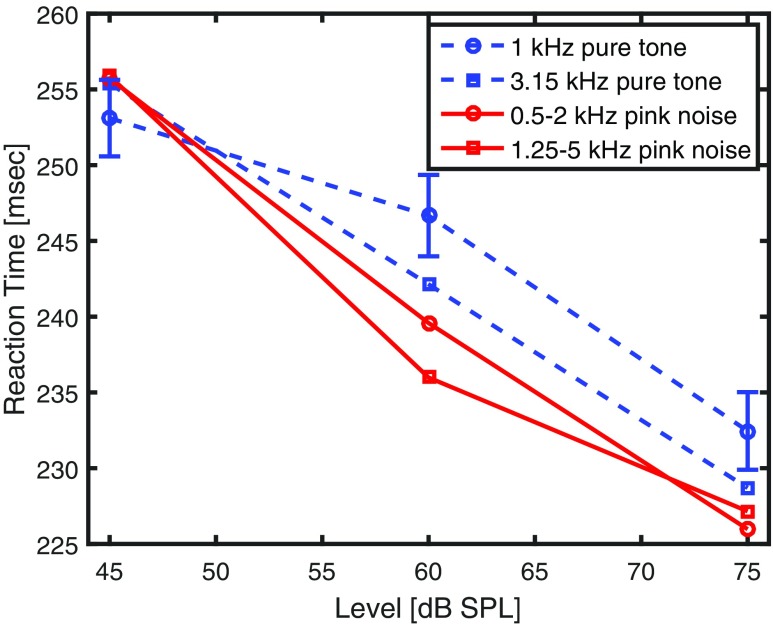
Results of Experiment 1: Geometric mean RTs as a function of sound pressure level to the onset of four sounds: 1-kHz pure tone (*blue circles*, *dashed line*), 3.15-kHz pure tone (*blue squares*, *dashed line*, 0.5 to 2 kHz bandpass-filtered pink noise (*red circles*, *solid line*), 1.25 to 5 kHz bandpass-filtered pink noise (*red squares*, *solid line*). Standard errors of the mean (SEM) are shown for the 1-kHz pure tone, one SEM into each direction. They are very similar for the other conditions

Geometric mean RTs, averaged across participants and trials, are shown in Fig. 1. Compared to other measures of central tendency, the geometric mean reduces the effect of longer RTs regarded as outliers. When looking at the data points at 60 dB SPL only, RTs are ordered as expected by the predictions of current loudness models. The 1-kHz pure tone, being the softest stimulus, shows the longest RT, followed by the 3.15-kHz pure tone, the mid-frequency noise and finally the high-frequency noise, which is the loudest sound. Even the magnitude of the effects appears to be reasonable, with the high-frequency noise at 60 dB SPL producing almost the same RT as the 1-kHz pure tone at 75 dB SPL, corresponding to the predicted 14-phon difference. The situation is similar at 75 dB SPL, with the 3.15-kHz pure tone, the mid-frequency noise and the high-frequency noise showing shorter RTs than the reference tone. However, the high-frequency noise does not show a shorter RT than the mid-frequency noise. At the lowest SPL of 45 dB, however, the 3.15-kHz pure tone and the two noises do not produce shorter RTs than the 1-kHz pure tone.

A 3 x 2 x 2 (SPL x bandwidth x frequency) within-subjects analysis of variance confirms these interactions with sound pressure level. Here, bandwidth refers to the discrimination of pure tone versus two-octave-wide noise and frequency to the lower ones versus the higher ones used. First of all, the main effect of sound pressure level is statistically significant, *F*(2,38) = 147, *p*< .001, ${\eta ^{2}_{p}}$ = .89, as is the main effect of bandwidth, *F*(1,19) = 6.27, *p*< .05, ${\eta ^{2}_{p}}$ = .25. No significant main effect of frequency was observed, *F*(1,19) = 1.07, *p* = .314, ${\eta ^{2}_{p}}$ = .053. There are two statistically significant interactions, of level with bandwidth, *F*(2,38) = 5.48, *p*< .01, ${\eta ^{2}_{p}}$ = .22, and of level with frequency, *F*(2,38) = 4.02, *p*< .05, ${\eta ^{2}_{p}}$ = .17.

### Discussion

When looking at the data for 60 and 75 dB SPL, it can be concluded that RT agrees with loudness in the region of moderate and higher sound pressure levels. At 60 dB SPL, the patterns of reaction time follow the predictions made by loudness models, showing the same order: 1-kHz pure tone, 3.15-kHz pure tone, mid-frequency noise, high-frequency noise (long to short, or soft to loud, respectively). They agree also quantitatively, with RTs showing a higher gain caused by the bandwidth of two octaves than the frequency shift towards the most sensitive area of human hearing, just like the loudness models predict. By contrast, Schlittenlacher et al., (2015) who used the method of adjustment and cross-modality matching with line length to assess loudness, found a similar loudness for the mid-frequency noise and high-frequency noise, suggesting just spectral summation and no additional frequency gain. At 75 dB SPL, the reaction times agree with these subjective loudness evaluations. As our earlier loudness study (Schlittenlacher et al., 2015) suggested, the effects of frequency and bandwidth do not sum in the expected manner, with the RTs to mid-frequency noise being very similar to those to the high-frequency noise.

The results are quite surprising at 45 dB SPL. It appears that the four experimental conditions are not distinguished at all. A possible explanation could be that at the lowest level used, RT is largely governed by the difficulty of detecting an onset, rather than being a correlate of loudness. Interestingly, Lentz et al., (2016) also suggested that the underlying mechanism governing RT depends on signal strength, leading to a distinction in architecture between RT models and accuracy models or loudness models. Potential discrepancies between RT and loudness at low levels with regard to an effect of frequency have been discussed in the literature: Although some studies suggest that RTs to tones of different frequencies follow the equal loudness contours pretty well in general (Chocholle 1940; Pfingst et al., 1975), Kohfeld et al. (1981) suggested that RTs correlate with the equal loudness contours at 60 and 80 phon, but not at 20 and 40 phon. However, they found a remarkably longer RT for the 1-kHz pure tone, which disagrees with the present study and has been critically commented before (Luce 1986, p. 70). Epstein and Florentine (2006) studied RT to 1-kHz and 4-kHz pure tones as a function of sensation level (SL). For very low SLs, they found inter-individual differences as some listeners had significantly shorter RTs to the 1-kHz pure tone while the opposite occurred for other listeners. Altogether, the present results might contribute to the evidence that RT does not mirror frequency effects of loudness at low levels, although it reasonably does so at higher levels.

More strikingly, the interaction between level and bandwidth, being caused by the lowest level studied, was not anticipated based on our previous loudness study (Schlittenlacher et al., 2015). By contrast, Wagner et al., (2004) found a good correlation between the LDERT and LDEL for sounds of different bandwidth throughout the range of levels reported, 20 to 100 dB SPL. Their broadband noise had the same cutoff frequencies as one of our stimuli, 500 Hz and 2 kHz. However, their second stimulus was a narrowband noise from 940 to 1064 Hz. There might be a difference caused by the different characteristics of a pure tone and a narrowband noise still having a bandwidth of 124 Hz.

To sum up, RT measurements correlate with calculated loudness quite well for sound pressure levels of 60 dB and higher, both for frequency and bandwidth effects. However, the interaction between level and bandwidth needs further consideration.

## Experiment 2: Broadband pink noise

### Stimuli and procedure

In order to study this interaction between sound pressure level and type of sound in more detail, Experiment 2 investigated two sounds only, but using a wider range of more finely graded sound pressure levels. These two sounds were a 1-kHz pure tone and pink noise ranging from 200 Hz to 20 kHz, leading to a comparison of the RTs to a pure tone and to a broadband noise spanning over 22 Bark (see Fastl and Zwicker 2007). The pure tone was presented at sound pressure levels ranging from 35 to 85 dB, the broadband noise from 25 to 75 dB, both in steps of 5 dB. The different sound pressure levels were chosen so that the range of loudness levels is similar and thus similar RTs are expected for the two kinds of sounds.

Each participant completed 60 trials for each of the 22 stimuli. They were collected in ten blocks, distributed over two sessions.

### Results

**Fig. 2:**
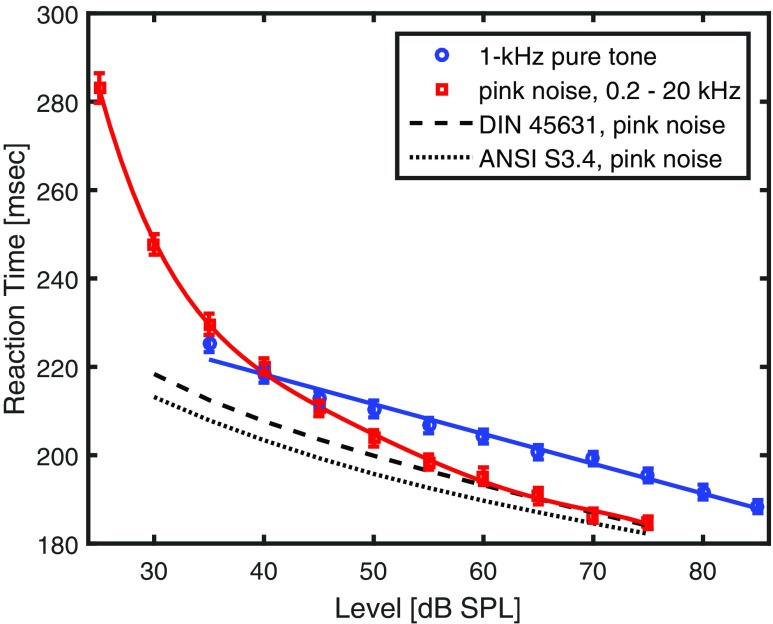
Results of Experiment 2: Geometric mean reaction times of 21 listeners as a function of sound pressure level for 1-kHz pure tones (*blue circles*) and pink noise (*red squares*). *Error bars* indicate one standard error of the mean into each direction. The *dashed line* shows predictions made by the DIN 45631 loudness model, using the distance to the regression line of the 1-kHz pure tone for equalizing the LDEL with the LDERT. The *dotted line* shows the same for ANSI S3.4-2007

The results are illustrated in Fig. 2 as a function of sound pressure level with red squares depicting RTs to the onset of pink noise and blue circles to 1-kHz pure tones. The RTs for the 1-kHz pure tones may be fitted by a linear regression with a slope decreasing with 0.67 msec per dB. Under the assumption that the LDEL corresponds to the LDERT, predictions for the pink noise condition made by loudness models may be compared to RTs by using the horizontal distance in dB to the regression line for the 1-kHz pure tone. This means that a predicted RT for pink noise can be deduced from loudness predictions by using the predicted level difference to the 1-kHz pure tone and actual RTs for the latter. That was done for DIN 45631 (1991, dashed line) and ANSI S3.4-2007 (dotted line). In general, both loudness models predict a similar relationship between the pure tone and pink noise in the range of levels studied, i.e. the pink noise needs considerably less intensity to produce the same loudness as a 1-kHz pure tone. However, they differ systematically, but not in principle, as ANSI S3.4-2007 predicts this LDEL to be about 5 dB larger than DIN 45631 suggests. The actual RTs for the pink noise were fitted by a polynomial of 5th order. The two regressions for the 1-kHz pure tone and pink noise cross at 40 dB SPL.

A 9 x 2 (SPL x sound) within-subjects analysis of variance over the SPLs studied for both kinds of sounds yields significant main effects of SPL, *F*(8,160) = 186, *p*< .001, ${\eta ^{2}_{p}}$ = .90 and sound, *F*(1,20) = 17.6, *p*< .001, ${\eta ^{2}_{p}}$ = .47. The interaction between SPL and sound is also significant, *F*(8,160) = 9.79, *p*< .001, ${\eta ^{2}_{p}}$ = .33.

### Discussion

When considering the results at 60 dB SPL and higher only, for which Experiment 1 suggested a good correlation between calculated loudness and RT, the actual RTs to the pink noise coincide excellently with the predictions made by the DIN 45631 loudness model. This implies these RTs also match well with subjective loudness evaluations since previous studies (Schlittenlacher et al., 2011; Schlittenlacher et al., 2015) have attested a good fit of this model regarding pink noise.

However, the statistical analyses confirm the interaction between the type of sound (tone or noise) and sound pressure level with a crossing around 40 dB SPL, which was not predicted by loudness models or subjective loudness evaluations in the range of levels studied (see also Zwicker 1958). RTs for the pink noise start to deviate from the model predictions or a linear regression (not shown in Fig. 2) around 50 to 55 dB SPL, with the discrepancy becoming larger the lower the sound pressure level gets. The question arises why RTs increase that much at the lowest levels studied. Kohfeld et al., (1981, Fig. 4) and Chocholle (1940, Fig. 1 to 3) measured RTs to the onset of 1-kHz pure tones as a function of intensity from near threshold in quiet to about 100 dB SPL. RT decreases approximately linearly with sound pressure levels of 30 or 40 dB and higher, but is considerably longer at lower levels with the slope becoming steeper and steeper towards the threshold in quiet. Figure 2 of the present work indicates a similar behavior for the 1-kHz pure tone, since its lowest level, 35 dB SPL, is the only one which lies more than a standard error of the mean above the regression line.

Altogether, and starting inspection from high levels downwards, the 1-kHz pure tone starts to deviate from a linear regression at around 35 dB SPL while the pink noise studied departs from the model predictions at around 50 to 55 dB SPL already. When looking at third-octave levels rather than absolute level, they are more similar. The range of the pink noise from 200 Hz to 20 kHz consists of 20 third octaves, thus, its third-octave level is 13 dB lower than its absolute level. These 13 dB are also obtained when dividing the bandwidth into 22 critical bands, for which third octaves are a good approximation. Thus, both the pure tone and the pink noise show a good linear relation between reaction time in milliseconds and sound pressure level in decibels for critical-band levels of 40 dB and higher.

It is interesting that the well-known loudness-intensity function is typically split into two regions as well. ISO 532 (1975) uses Stevens’ power law (Stevens 1957) for the conversion between phon and sone above 40 phon, but a different and steeper function below. Florentine and Epstein (2006) propose an improved loudness-intensity function, consisting of two functions being split at 40 dB SPL. Thus, the interaction between level and sound found at low levels and the good correspondence of RT with loudness at higher levels might fit well to a reasoning with a different behavior of RT in two regions being split at a critical-band level of approximately 40 dB.

Furthermore, it should be considered that the mechanism of spectral summation might differ for RT and loudness. Loudness models usually integrate over specific loudness, i.e., loudness is summed across critical bands. RT models that take several parallel channels into account often are race models, i.e., a reaction is triggered when the first of several racers has reached the decision center. That effect is called statistical facilitation, as a faster arrival becomes more likely when more channels are involved. Originally thought for inputs of more than one modality (Raab 1962), such parallel processing models have been developed to be applicable for one modality only as well (Miller and Ulrich 2003). The principles of their parallel grains model (PGM), whereby a “grain” represents an information channel, conform well to modelling auditory processing with critical bands.

Applied to the present results, statistical facilitation covaries with spectral summation at higher sound pressure levels. As does the summation of specific loudness, it overcompensates for the effect that the loudness or level of a single critical band is smaller. That is different at low SPLs below 40 dB because of the strong increase of RT as a function of intensity towards threshold. In that case, statistical facilitation cannot compensate for the much slower “racers” anymore. In line with this reasoning, Schlittenlacher and Ellermeier (2015, Fig. 7) presented a relation between SPL and the activation time for a racer in the PGM, which was based on the data of several experiments. This relation is roughly linear for SPLs higher than 40 dB SPL, but its slope increases sharply towards lower levels.

Although our reasoning based on critical-band levels and differences in spectral summation can account for the present results, it must be noted that it cannot explain the differing results of Wagner et al., (2004, Fig. 5), where RT reflected loudness very well in the entire range of levels studied, i.e., including levels as low as 20 dB SPL. Their narrowband noise also has a bandwidth of less than a critical band. That is why it must be kept in mind that the reason for the interaction between sound pressure level and type of sound found in the present experiment might alternatively be explained by other aspects referring to the different nature of a pure tone and a noise. As the RTs to the broadband noise have a very similar shape in the present study and in Fig. 2 of Wagner et al. (2004), it might be that the different conclusions arise because of the differences between a pure tone and a narrowband noise.

## Experiment 3: Binaural effects

The same way Experiment 1 studied the combination of bandwidth and frequency effects, Experiment 3 shall investigate the combination of bandwidth and binaural effects. Edmonds and Culling (2009) had shown that the binaural gain for noise depends on the interaural correlation. Though the additional gain for uncorrelated noise appears to be small, about 2 dB for low-frequency narrowband noise and even smaller for other types of noises, that is a considerable amount for the binaural gain which is about 6 dB in their study. Furthermore, the present experiment shall scrutinize whether the binaural gain is the same for broadband noise as it is for 1-kHz pure tones. Previous work using RT as the dependent variable found the latter to be 5 to 6 dB (Schlittenlacher et al., 2014).

### Stimuli and procedure

All stimuli were white noise with cutoff frequencies of 20 Hz and 20 kHz. As the focus was on broadband sounds, this broad bandwidth was chosen instead of a narrow bandwidth covering low frequencies only, for which the effect of interaural correlation should be larger. The stimuli were presented at three sound pressure levels, 45, 60, and 75 dB and four aural presentation modes (APMs). These were a monaural presentation to the left ear only, monaural presentation to the right ear only, diotic presentation using the same white noise for both ears and uncorrelated binaural presentation using two different samples of white noise. In order to avoid a potential effect of the specific noise sample chosen, these two samples were frozen noise and each of the two was used in half of the right-ear, left-ear or diotic conditions, respectively.

Each participant contributed 50 trials to each of the twelve conditions. Data collection was distributed over five blocks.

### Results

**Fig. 3:**
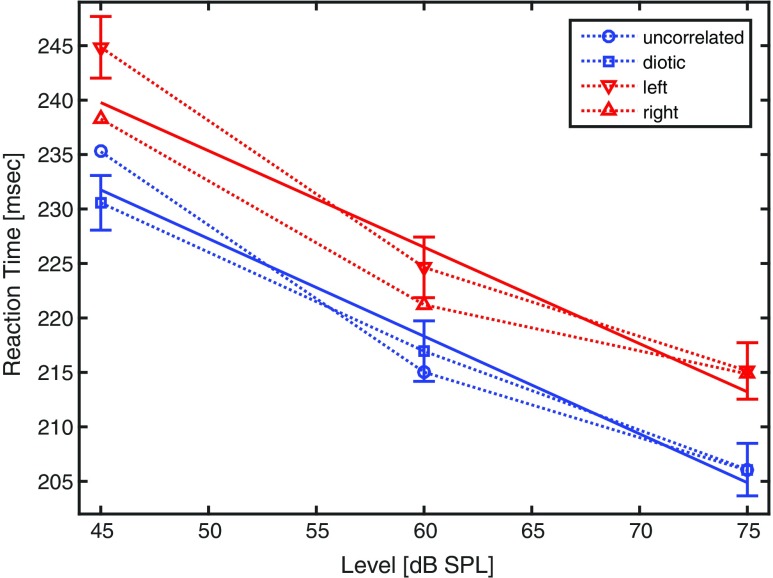
Results of Experiment 3: Geometric mean reaction times of 20 listeners to the onset of white noise as a function of sound pressure level (*abscissa*) and aural presentation mode (*blue squares*: diotic; *blue circles*: uncorrelated binaural; *red triangles pointing downwards*: left ear; *red triangles pointing upwards*: right ear). Linear regression lines were fitted to the binaural and monaural conditions. Standard errors of the mean are shown for diotic and right-ear conditions

Figure 3 illustrates the geometric mean RTs based on 1000 trials (20 participants x 50 trials) for each condition. There seems to be no difference between diotic and uncorrelated presentations, which produce virtually the same RT at 75 dB SPL with the uncorrelated noise resulting in a somewhat shorter RT at 60 dB SPL but a longer RT at 45 dB SPL. RTs in the monaural conditions are systematically longer than in the binaural ones. Furthermore, reactions to the right ear are faster than those to the left ear at two of the three levels studied.

A 3 x 4 (SPL x APM) within-subjects analysis of variance yields a significant main effect of SPL, *F*(2,38) = 150, *p*< .001, ${\eta ^{2}_{p}}$ = .89 and a significant main effect of APM, *F*(3,57) = 26.2, *p*< .001, ${\eta ^{2}_{p}}$ = .58. There is no statistically significant interaction between SPL and APM, *F*(1,19) = 1.50, *p* = .184, ${\eta ^{2}_{p}}$ = .07, implying that the decrease of RT with level is rather similar for all APMs. Two post hoc analyses of variance including the two binaural conditions only or the two monaural conditions only, respectively, yield no statistically significant difference between diotic and uncorrelated presentation, *F*(1,19) = 0.33, *p* = .570, ${\eta ^{2}_{p}}$ = .02. The difference between right and left ear is statistically significant, *F*(1,19) = 5.58, *p*< .05, ${\eta ^{2}_{p}}$ = .23.

Averaging across the two monaural and the two binaural conditions yields a difference between binaural and monaural presentation of 7 to 9 ms. Though the three levels might not be sufficient to test whether the relation between RT and SPL is linear, linear regression lines (solid lines in Fig. 3) might be suitable to express the binaural gain in decibels. The two regression lines are almost parallel, having slopes of -0.90 or -0.89 ms per dB, respectively, and a horizontal distance of 9 dB.

### Discussion

The present results do not show an additional binaural gain for uncorrelated noise. Although the effect is expected to be small for the white noise used, even the descriptive results, being based on 1000 trials for each data point, do not show any such tendency, thus being at odds with the loudness matches reported by Edmonds and Culling (2009). Maybe the effect is considerably smaller than the standard errors of the mean. That is consistent with the conclusions drawn by Edmonds and Culling that the effect diminishes if the noise contains high-frequency components.

By contrast, the binaural gain between monaural and binaural presentation is clearly visible. Its magnitude of 9 dB, obtained by a linear fit, is somewhat surprising as the majority of newer studies exhibit a smaller binaural gain up to 6 dB (see Sivonen & Ellermeier 2011 for a review). However, it is in the range of the binaural-to-monaural loudness evaluations conducted by Whilby et al., (2006), who suggest a level-dependent binaural gain of up to 10 dB. As a side result, the right ear advantage, presumably being caused by ipsilateral advantages and the fact that most participants responded with their right hand, was confirmed (see Schlittenlacher 2014).

Altogether, the binaural gain can be observed not only in loudness evaluations, but also in RT measurements. It amounts to 5 to 6 dB for 1-kHz pure tones (Schlittenlacher et al., 2014) and is somewhat higher for white noise, around 9 dB.

## General discussion

Simple reaction time was shown to correlate strongly with loudness and reflecting known effects of bandwidth if the sounds used have sound pressure levels of 60 dB or higher. In that range of levels, RT measurements mirror the amount of spectral loudness summation very well. The level difference required for equal reaction time between 1-kHz pure tones and pink noise matches the level difference required for equal loudness determined in recent studies almost perfectly (compare Exp. 2 with Schlittenlacher et al., 2011). The RT results are even somewhat closer to loudness model predictions than the loudness matches are, though they cannot explain whether this originates from a procedural bias in the loudness matches or rather an inaccuracy in both calculated loudness and RT measurements. Furthermore, the effects of spectral summation and the increased sensitivity around 3.15 kHz add up very similarly in RT and loudness experiments (compare Exp. 1 with Schlittenlacher et al., 2015). However, these statements do not hold for lower sound pressure levels in the present experiments. Spectral summation or statistical facilitation does not lead to shorter reaction times for pink noise at those levels. Reasons might be found in the combination of lower critical-band levels and a steep increase of RT at low levels. Discrepancies with respect to the RT study of Wagner et al., (2004), who did not find such an interaction, might be explained by the different characteristics of the narrowband noise they used compared to a 1-kHz pure tone used in the present experiments. However, when RT measurements are employed to measure loudness, the comparison with a 1-kHz pure tone is important since it is the reference tone.

Lentz et al., (2016) tested which type of model is most suitable to predict RTs by not only analyzing means but also survivor functions and hazard functions. They found race models to be more likely than summation models or other candidates to explain the underlying RT distributions. Furthermore, they found their results to be in the limited capacity range (see Townsend and Wenger 2004), implying longer RTs for a two-tone complex than a standard parallel model would suggest. This is in line with our reasoning of a duality between race models for RT and summation models for loudness, and also supports the need for more complex race models such as the “parallel grains model” (Miller and Ulrich 2003), for example.

Investigating the binaural level difference required for equal reaction time in the case of white noise (Exp. 3) led to a comparatively large binaural LDERT of 9 dB. This is 3 to 4 dB greater than previous RT studies determined for a 1-kHz pure tone (Schlittenlacher et al., 2014), however, it is still within the range of levels which loudness studies yield for the binaural LDEL, though towards the upper end.

Altogether, the present work suggests that RT reflects the bandwidth effects known from studies on loudness, also in combination with frequency effects or binaural summation. Experiment 3 showed that the effects of binaural summation and spectral summation lead to a binaural gain that is in the range of that found in loudness studies. The LDERTs found in Experiments 1 and 2 agreed with LDELs predicted by loudness models for moderate and high sound pressure levels. Furthermore, the effects of frequency and spectral summation combine as expected from loudness models and studies at these levels. However, RT to broadband noises is longer than that to pure tones at low levels, suggesting that the underlying mechanism of RT is not just a simple correlate of loudness.
